# The Neurotropic Parasite *Toxoplasma gondii* Induces Astrocyte Polarization Through NFκB Pathway

**DOI:** 10.3389/fmed.2019.00267

**Published:** 2019-11-19

**Authors:** Yu Jin, Yong Yao, Saeed El-Ashram, Jiaming Tian, Jilong Shen, Yongsheng Ji

**Affiliations:** ^1^Anhui Provincial Laboratory of Microbiology and Parasitology, Laboratory of Tropical and Parasitic Diseases Control, Department of Microbiology and Parasitology, Anhui Medical University, Hefei, China; ^2^School of Life Science and Engineering, Foshan University, Foshan, China; ^3^Faculty of Science, Kafrelsheikh University, Kafr El-Shaikh, Egypt

**Keywords:** *Toxoplasma gondii*, encephalitis, astrocyte, NFκB pathway, neuron

## Abstract

**Background:** *Toxoplasma gondii* is a protozoan parasite that chronically infects nearly one-third of the world's human population. In immunosuppressed individuals and fetus, infection with *T. gondii* contributes to a series of devastating conditions, including toxoplasmic encephalitis (TE), which is characterized by neuron damage in the central nervous system (CNS). Astrocyte polarization is currently found in some neurodegenerative diseases, and A1 subtype of astrocyte leads to neuron apoptosis. However, little information has been available on the role of astrocyte polarization in TE.

**Methods:** In the present study, we established a mouse model to study TE and detected A1 astrocyte in the brains of mice with TE. Expression level of A1 astrocyte-specific marker C3 was evaluated using indirect fluorescent assay (IFA) and Western blotting. Primary mouse astrocytes were incubated with different concentrations of *T. gondii* excreted-secreted antigens (*Tg*ESAs) *in vitro*. Expression level of C3 and A1 astrocyte-specific transcription levels were assessed using Western blotting and qRT-PCR, respectively. Bay11-7082 was used to study nuclear factor (NF) κB pathway in *Tg*ESA-induced astrocyte polarization.

**Results:** In mice with TE, the proportion of A1 astrocyte (GFAP^+^C3^+^) increased significantly. The results of *in vitro* study showed that *Tg*ESAs induced astrocyte polarization to A1 subtype. Blocking of NFκB pathway by Bay11-7082 inhibited *Tg*ESA-induced astrocyte polarization.

**Conclusions:** Our preliminary study showed the involvement of A1 astrocyte in the process of TE in mice, and *Tg*ESAs could trigger astrocyte to polarize to A1 subtype. These findings suggest a new mechanism underlying the neuropathogenesis induced by *T. gondii* infection.

## Introduction

*Toxoplasma gondii* is an obligate intracellular protozoan parasite that chronically infects the central nervous system (CNS) of up to one-third of the human population in the world (1). Humans get infected with such disease by ingesting water or food contaminated with oocysts shed by cats or consumption of raw or undercooked meat containing a tissue cyst or congenitally by transplacental transmission of tachyzoites (2). Upon infection with *T. gondii*, fast-replicating tachyzoites infect a wide range of host cells, including neurons. Tachyzoites convert into slow-replicating bradyzoites, which persist as cysts in neurons under the pressure of the immune response.

Although most infected persons show no clinical symptoms, chronic *T. gondii* infection could impair host neuron function and structure (3–5), which may alter the behavior of humans or even increase the risk for neurodegenerative and psychiatric disorders (6, 7). In the developing fetus and immunocompromised individuals, such as AIDS patients or organ transplant recipients, *Toxoplasma* infection can cause a devastating neurologic disease. Symptomatic brain infection with *Toxoplasma* is known as toxoplasmic encephalitis (TE) and can clinically present with dizziness, headaches, and seizures. Currently, TE occurs in untreated or undiagnosed AIDS patients and in patients on new immunomodulants (8). In TE, *T. gondii* bradyzoites within cysts switch to tachyzoites, which infect and destroy brain-resident cells. Previous *in vitro* and *in vivo* evidences suggest that neurons serve as primary target cells for *T. gondii* tachyzoites and bradyzoites (1, 9).

An *in vitro* culture of neurons with *T. gondii* tachyzoites at a low multiplicity of infection (MOI) as previously described (10) induced the formation of a large cyst rather than the lysis of neurons. However, the *in vivo* TE mouse model showed that the neuronal damage was increased in the brain, and *T. gondii* infection induced activated microglia, which contributed to neuronal apoptosis (11). In addition, *T. gondii* excreted-secreted antigens (ESAs) induce apoptosis of the neural stem cells (NSCs) through endoplasmic reticulum stress (ERS) signaling pathways and inhibit differentiation of C17.2 neural stem cells through Wnt/β-catenin signaling pathway (12, 13). Whether other CNS resident cells are involved in neuron loss in TE is still an enigma.

Astrocytes are the most common glial cells within the cerebral cortex, which provide trophic support for neurons, promote formation and function of synapses, and prune synapses by phagocytosis (14–16). These cells also perform a diversity of functions, including participation in the immune response of the brain and undergo a pronounced transformation called reactive astrocytosis after brain injuries and neurodegenerative diseases (17).

Recent studies have demonstrated that proinflammatory microglia induce the formation of a subtype of astrocytes (termed A1 astrocytes), which are characterized by highly upregulated classical complement cascade genes (i.e., C3) shown to be destructive to synapses and are strongly neurotoxic and rapidly kill neurons (18). A1 astrocytes are abundant in various human neurodegenerative diseases, including Alzheimer's disease, Huntington's disease, Parkinson's disease, amyotrophic lateral sclerosis, and multiple sclerosis (18). A1-like astrocyte reactivity is induced in normal aged brains that are vulnerable to injury and cognitive function declines (19). However, whether *T. gondii* infection induces astrocyte polarization to A1 and the role of A1 astrocytes in neuron death in TE are still not clear. In the present study, we aimed to investigate the effects of the ESAs of *T. gondii* (*Tg*-ESAs) on astrocyte polarization and assess the involvement of nuclear factor (NF) κB signaling pathway in *Tg*-ESAs-induced astrocyte polarization. This study provides insight into the underlying molecular mechanisms that regulate neuropathogenesis in TE.

## Materials and Methods

### Cell and Parasite

*T. gondii* Wh6 strain (avirulent strain) with genotype Chinese 1 (ToxoDB#9) was isolated as previously described (20). Cysts were maintained in the brain of chronically infected mice for *in vivo* infection. To collect cysts, brains from infected mice were mechanically homogenized in 1-ml sterile phosphate-buffered saline (PBS). Cyst numbers were counted in a 10-μl brain suspension using a light microscope (21). Tachyzoites of *T. gondii* were passaged in human foreskin fibroblast (HFF) monolayers for *in vitro* experiments. Mouse primary astrocytes were purchased from FenghuiShengwu (Changsha, China) and cultured in Dulbecco's modified Eagle medium (DMEM) medium supplemented with 10% fetal bovine serum.

### Mice and Infection

Mice were divided into three groups (three mice/group): control group (non-infection group), chronic group (chronic infection without cyclophosphamide treatment), and TE group (chronic infection with cyclophosphamide treatment). The Wh6 strain cysts were prepared by homogenization of the brain tissues in phosphate-buffered saline (PBS). Seven-week-old female BALB/c mice were intragastrically administered with 30 cysts. After 6 weeks, mice with latent infection were intraperitoneally injected with cyclophosphamide (50 mg/kg; Baxter Oncology GmbH, Germany) to induce recurrence of toxoplasmosis as previously described (11). Seven days later, all mice of the three groups were euthanized for collection of the brain tissues for further experiments. All experimental procedures were approved by the Institutional Animal Care and Use Committee of Anhui Medical University.

### Treatment of Astrocyte With *Tg*ESAs

ESAs from *T. gondii* were prepared as described previously (12). Tachyzoites of *T. gondii* were harvested as described above.

After resuspension with serum-free DMEM, 2 × 10^7^ freshly collected tachyzoites were added into HFF monolayers. *T. gondii*-infected HFFs were further cultured in the serum-free DMEM medium at 37°C in 5% CO_2_ for another 48 h. The supernatants of the infected HFFs were collected by centrifugation at 12,000 g for 10 min at 4°C and then filtered through a 0.22-mm membrane filter. Protein concentration in the supernatants was determined by BCA kits according to the manufacturer's instructions (Thermo-Fisher, Boston, MA). Protein samples were stored at −80°C until use. Non-infected HFFs in serum-free DMEM were used as a negative control.

Mouse primary astrocytes were seeded in six-well cell culture plates. Cells were treated with different doses of *Tg*ESAs (0, 0.10, 0.15, and 0.30 mg/ml) for 24 h when the cell confluence reached approximately 70%. Then, C3 protein expression level and A1-specific gene transcription levels were detected by Western blotting and quantitative real-time PCR (qRT-PCR), respectively. In some experiments, astrocytes were pretreated with NFκB inhibitor BAY11-7082 (1 and 5 μM) (22). After 12 h, *Tg*ESAs (0.30 mg/ml) was added, and cells were co-cultured for further 24 h.

### Immunofluorescence Assays

Mice were anesthetized with 1% pentobarbital and transcardially perfused with 20 ml ice-cold 4% paraformaldehyde after an initial flush with 20 ml ice-cold 0.01 M PBS. Brains were removed and post-fixed with 4% paraformaldehyde for 12 h. Brain tissues were subsequently dehydrated in 30% sucrose in 0.01 M PBS for 48 h. Tissues were embedded in optimal cutting temperature compound (OCT Compound, SAKURA, USA) and then sliced coronally (10–20 μm) on a cryostat microtome (CM3050S, Leica, Germany). For immunofluorescence staining, the samples were blocked with 5% bovine serum albumin (BSA) and 0.5% Triton X-100 (containing 0.02% normal goat serum) for 2 h at room temperature. The samples were incubated with primary antibodies overnight at 4°C and then with the appropriate fluorescent secondary antibodies for 2 h at room temperature. Primary antibodies included anti-GFAP (1:50, Abcam) and anti-C3 (1:400, Abcam). Fluorescent images (astrocytes in mouse cortex) were captured using an Olympus BX53 fluorescence microscope (Olympus, Tokyo, Japan) and processed using ImageJ software (ImageJ, National Institutes of Health, Bethesda, MD) for quantification of florescence intensity.

### ELISA

Mouse brain tissues (mainly from cortex, 100 mg) were homogenized intensively and centrifuged at 12,000 g for 15 min at 4°C. Concentrations of tumor necrosis factor (TNF)-α and interleukin (IL)-1α in the mouse brain were evaluated using commercial kits according to the manufacturer's instructions (BioLegend, USA).

### Western Blotting

Proteins extracted from mouse brains (mainly from cortex) or astrocytes were separated using SDS-PAGE electrophoresis and then transferred to a PVDF membrane (Millipore, USA). After blocking with 5% BSA for 1 h at room temperature, PVDF membrane was incubated with primary antibodies overnight at 4°C and then with the fluorescent secondary antibodies for 1 h at room temperature. Primary antibodies included anti-GFAP (1:1,000, Abcam), anti-C3 (1:2,000, Abcam), anti-β-actin (1:5,000, Abcam), and anti-Neu-N (1:1,000, Cell Signaling Technology). Fluorescent images were captured by the Tacon 5200 (Biotanon, China) and analyzed using ImageJ software.

### Quantitative Real-Time PCR Assay

Total RNA was extracted from astrocytes using Trizol reagent (Tiangen Biotech, China) according to the manufacturer's protocols. The concentrations of the extracted RNA were measured using NanoDrop 2000c (ThermoFisher, USA) (23–25). Total RNA (1 μg) was reverse-transcribed to cDNA using a reverse transcription kit (TaKaRa, Japan). QRT-PCR was performed on the QuantStudio® 6 Flex real-time PCR instrument (Applied Biosystems, USA) using SYBR™ Green qPCR Master Mix (ThermoFisher, USA). Gene expression levels were normalized to β-tubulin levels using the 2^−ΔΔ*Ct*^ method. All primers for A1 astrocyte-specific genes used in the present study are listed in Table 1 (18).

**Table 1:** Primers used for quantitative real-time PCR in the present study.

**Gene**	**Forward**	**Reverse**	**Length (bp)**
***Amigo2***	GAGGCGACCATAATGTCGTT	GCATCCAACAGTCCGATTCT	263
***Fbln5***	CTTCAGATGCAAGCAACAA	AGGCAGTGTCAGAGGCCTTA	281
***Ggta1***	GTGAACAGCATGAGGGGTTT	GTTTTGTTGCCTCTGGGTGT	115
***H2-D1***	TCCGAGATTGTAAAGCGTGAAGA	ACAGGGCAGTGCAGGGATAG	204
***H2-T23***	GGACCGCGAATGACATAGC	GCACCTCAGGGTGACTTCAT	212
***Iigp1***	GGGGCAATAGCTCATTGGTA	ACCTCGAAGACATCCCCTTT	104
***Psmb8***	CAGTCCTGAAGAGGCCTACG	CACTTTCACCCAACCGTCTT	121
***Serping1***	ACAGCCCCCTCTGAATTCTT	GGATGCTCTCCAAGTTGCTC	299

### Statistical Analysis

All statistical analyses were performed using SPSS (Version 24, IBM, USA). All data are expressed as mean ± SEM (standard error of the mean). Differences between groups were assessed by one-way ANOVA followed by Student-Newman-Keuls (SNK) multiple comparison posttest or Student's *t* test. Differences were considered statistically significant when *P* < 0.05.

## Results

### Establishment of a Murine Model of TE

**Figure 1:**
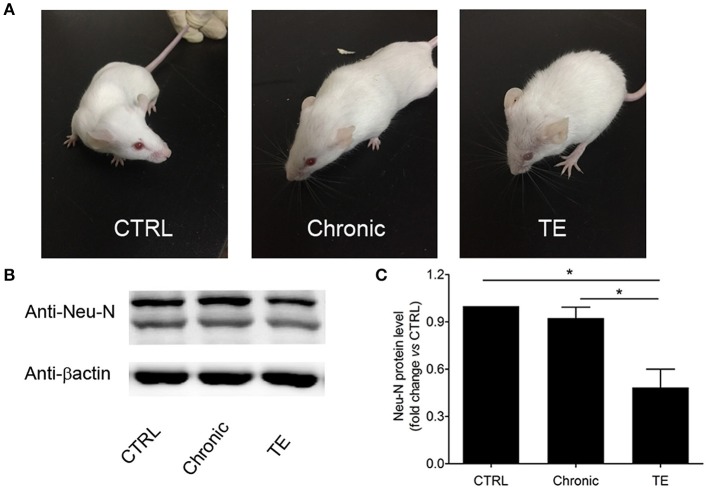
Establishment of a murine model of toxoplasmic encephalitis (TE) **(A)** and identification of neuron damage in the brain of mice with TE **(B,C)**. *P < 0.05.

To establish a mouse model of TE, BALB/c mice were orally infected with Wh6 tissue cysts. Six weeks later, *Toxoplasma*-infected mice were immunosuppressed by intraperitoneal injection with cyclophosphamide to reactivate chronic *T. gondii* infection. As we can see in Figure 1A, mice with TE showed piloerection and hunching posture, which are typical physical characteristics of acute toxoplasmosis. Western blotting results showed that expression levels of neuron marker Neu-N in mice with TE decreased significantly compared to those in the control group (Figures 1B,C, Ctrl group vs. TE group, 1.00 ± 0.00 vs. 0.52 ± 0.09, *P* < 0.05). These results indicated that neurons in the central nervous system (CNS) were damaged when the mouse brains were infected with tachyzoites of *T. gondii*.

### Astrocyte Polarization to A1 in the Mouse Brain With TE

**Figure 2:**
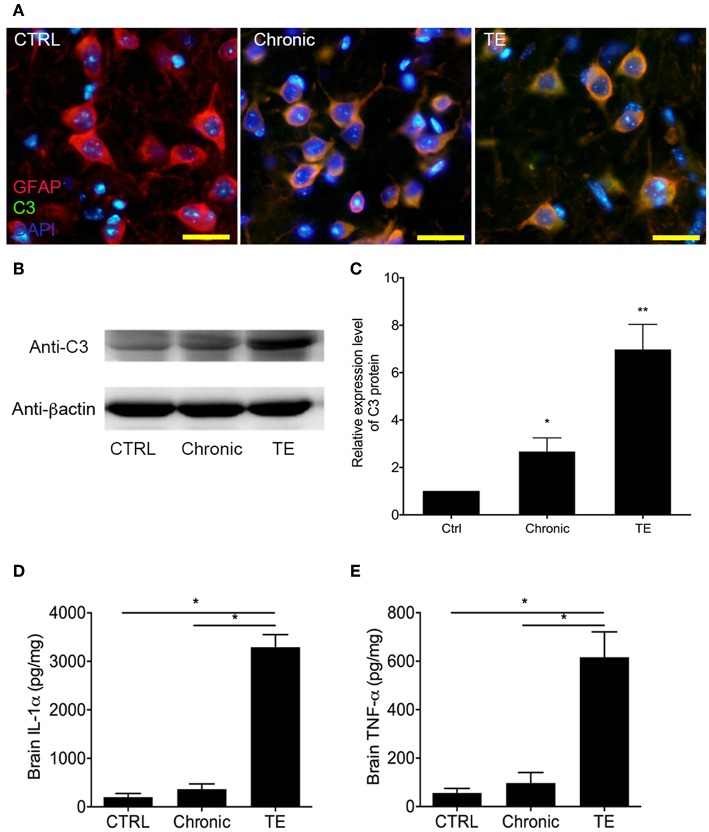
Identification of A1 astrocyte (GFAP^+^C3^+^) in the brain of mice with toxoplasmic encephalitis (TE) using indirect fluorescent assay (IFA) **(A)** and Western blotting analysis of C3 expression level in the mouse brain **(B,C)**. Evaluation of interleukin (IL)-1α and tumor necrosis factor (TNF)-α in the mouse brain using commercial ELISA Kits **(D,E)**. Bar, 50 μm, *P < 0.05 vs. control group.

The proportion of A1 astrocyte (GFAP^+^C3^+^) was significantly higher in the TE group compared to the controls (Figure 2A) as detected by immunofluorescent assay (IFA).

The expression level of A1-specific protein C3 was evaluated using Western blotting. Consistent with IFA result, the expression level of C3 in brains of the TE group increased dramatically (Figures 2B,C, Ctrl group vs. TE group, 1.00 ± 0.00 vs. 6.98±1.06, *P* < 0.05), although the expression level of C3 in the chronic group was apparently higher than that of the control group. The concentrations of A1 cytokine inducers (TNF-α and IL-1α) were subsequently measured in the mouse brain using ELISA. The results indicated that TNF-α (Ctrl group vs. TE group, 56.20 ± 19.49 vs. 616.4 ± 104.8, *P* < 0.05) and IL-1α (Ctrl group vs. TE group, 198.2 ± 75.34 vs. 3,291 ± 260.0, *P* < 0.05) expression levels were remarkably enhanced in mice with TE (Figures 2D,E).

### *Tg*ESAs Induced Astrocyte Polarization to A1 *via* the NFκb Pathway

**Figure 3:**
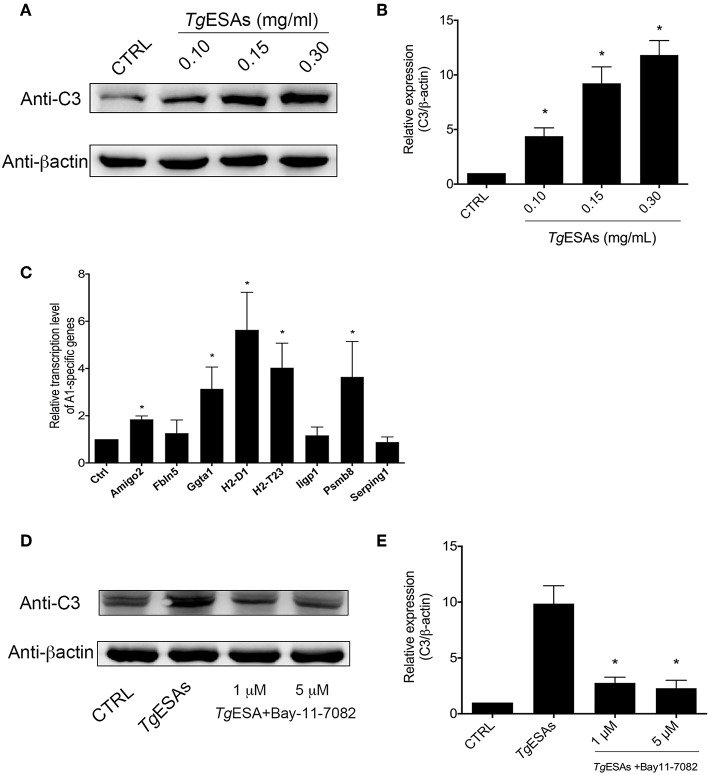
*T. gondii* excreted-secreted antigen (*Tg*ESA) induction of astrocyte to A1 subtype *via* nuclear factor (NF)κB pathway. **(A,B)** *Tg*ESA treatment increased C3 expression level of astrocyte in a dose-dependent manner. **(C)** *Tg*ESA treatment changed A1-specific gene transcript levels. **(D,E)** Blockage of NFκB pathway inhibited *Tg*ESA-induced expression of C3 of astrocyte. *P < 0.05 vs. CTRL in **(B,C)**; *P < 0.05 vs. *Tg*ESAs in **(E)**.

Since *T. gondii* tachyzoites can manipulate cells in the mouse brain that they do not productively invade (26), the non-colocalization of tachyzoites and A1 astrocytes prompted us to hypothesize that *Tg*ESAs induced astrocyte polarization. To test this hypothesis, the mouse primary astrocyte was incubated *in vitro* with *Tg*ESAs. Western blotting results showed that the expression level of C3 was robustly elevated in the *Tg*ESA group (Ctrl group vs. 0.30 mg/ml *Tg*ESA group, 1.00 ± 0.00 vs. 11.83 ± 1.32, *P* < 0.05) (Figures 3A,B). Then, the transcription levels of A1-specific genes were evaluated using qRT-PCR (18). As shown in Figure 3C, after incubation with *Tg*ESAs, the transcription levels of *Amigo2, Ggta1, H2-D1, H2-T23*, and *Psmb8* genes were enhanced apparently, while there was no statistical difference in the transcription levels of *Fbln5, Iigp1*, and *Serping1* genes between the control and *Tg*ESA groups. Interestingly, when primary astrocytes were pretreated with NFκB inhibitor BAY11-7082, the C3 expression level decreased significantly comparing to the non-inhibitor treatment group (*Tg*ESA group). Differences in the C3 expression level between the control and BAY11-7082 groups (*Tg*ESA group vs. *Tg*ESA + 5 μM BAY group, 9.87 ± 1.60 vs. 2.30 ± 0.70, *P* < 0.05) suggested that *Tg*ESAs polarized astrocytes to A1 subtype *via* NFκB signaling pathway (Figures 3D,E). These results indicated that *Tg*ESAs induced mouse primary astrocyte polarization to A1 subtype *in vitro* through activation of NFκB signaling pathway.

## Discussion

Reactivation of chronic *T. gondii* infection can cause life-threatening TE in immunocompromised individuals. Here, we show for the first time that *T. gondii* infection in CNS leads to astrocyte polarization to A1 subtype, which potentially harms neurons.

A1 astrocytes have been reported in the lipopolysaccharide (LPS)-induced CNS inflammation model (18), traumatic brain injury (TBI), and prion diseases (27, 28). In murine models of TE, astrocyte activation and proliferation are prominent, and these cells produce chemokines that can influence the recruitment of T cells and dendritic cells (DCs) as well as microglial cell activation (29). In this study, we found that the majority of activated astrocytes in mice with TE were A1 subtype (C3 positive), indicating that activated astrocytes may function as a double-edged sword in the development of TE. A1 subtype can act as chemokine producers for inflammatory cell recruitment. Additionally, A1 subtype may also release unidentified chemicals toxic for neurons, which needs to be addressed in further studies.

A previous study has demonstrated reported that activated microglia secrete IL-1α, TNF-α, and C1q in LPS-induced astrocyte polarization. These cytokines were essential for astrocyte polarization to A1 subtype (18). In the present study, increased expression levels of IL-1α and TNF-α were observed. Furthermore, IL-1α and TNF-α could be produced by mouse CNS-resident immune cells, such as CD11b^+^ microglia cells, since mice were immunosuppressed using cyclophosphamide. The cellular origin of these two cytokines and the cross talk between microglia and astrocytes should be determined in future studies.

A previous report demonstrated that the expression levels of cerebral cortical C1q were significantly elevated during *T. gondii* chronic infection (30). In this study, we found that the expression level of C3 was enhanced in acute TE. Based on our *in vitro* experiment results, the exposure of astrocyte to *Tg*ESAs may contribute to the enhancement of C3. C1q and C3 were mainly expressed by activated astrocytes. We can hypothesize that *T. gondii* chronic infection causes host behavioral changes partially through C1q activation and interaction with bradyzoite cysts. If bradyzoite cysts were disrupted, egressed parasites secrete effector proteins to trigger A1 astrocytes (C3^+^) resulting in TE. Thus, it is worthy to further study the detailed mechanism of how astrocyte shifts its expression from C1q to C3 at different stages of *T. gondii* infection.

In spinal cord injury (SCI), exosomes derived from mesenchymal stem cells (MSCs) reduced A1 astrocytes *via* downregulation of NFκB pathway (31). Similarly, in this study, we found that inhibition of NFκB pathway by BAY11-7082 significantly reduced the *Tg*ESA-induced C3 expression level in astrocytes. *T. gondii*-derived profilin recognized macrophage TLR-11 and induced the expressions of macrophage chemotactic protein 1 (MCP-1), IL-12, and interferon gamma (IFN-γ) through NFκB activation (32). The culture supernatant of *T. gondii* may inhibit THP-1 cell and arrest the cell cycle of THP-1 cells at G_0_/G_1_ phase mainly by regulating the expression of gene NFκB, cyclin D1 (33), while ROP16 of *T. gondii* could regulate NFκB pathway of A549 cells (34). The effector proteins in *Tg*ESAs linking NFκB activation and astrocyte plasticity need to be determined in further experiments.

In summary, we reported for the first time that neurotoxic A1 astrocytes were involved in TE, and *Tg*ESAs induced astrocyte polarization through NFκB activation. Our results provide new insights into the role of resident cells in the neuropathogenesis in brain toxoplasmosis.

## Data Availability Statement

The datasets generated for this study are available on request to the corresponding author.

## Ethics Statement

The animal study was reviewed and approved by the Institutional Animal Care and Use Committee of Anhui Medical University.

## Author Contributions

YJin, YY, and JT performed the experiments and analyzed the data. SE-A and YJi wrote the manuscript. YJi and JS designed the study.

### Conflict of Interest

The authors declare that the research was conducted in the absence of any commercial or financial relationships that could be construed as a potential conflict of interest.
