# Corrigendum: The Neurotropic Parasite *Toxoplasma gondii* Induces Astrocyte Polarization Through NFκB Pathway

**DOI:** 10.3389/fmed.2019.00299

**Published:** 2019-12-20

**Authors:** Yu Jin, Yong Yao, Saeed El-Ashram, Jiaming Tian, Jilong Shen, Yongsheng Ji

**Affiliations:** ^1^Anhui Provincial Laboratory of Microbiology and Parasitology, Laboratory of Tropical and Parasitic Diseases Control, Department of Microbiology and Parasitology, Anhui Medical University, Hefei, China; ^2^School of Life Science and Engineering, Foshan University, Foshan, China; ^3^Faculty of Science, Kafrelsheikh University, Kafr El-Shaikh, Egypt

**Keywords:** *Toxoplasma gondii*, encephalitis, astrocyte, NFκB pathway, neuron

In the original article, there was a mistake in Figure 1 as published. We used the incorrect image for the reference protein in Figure 1B. The corrected Figure 1 appears below.

**Figure 1:**
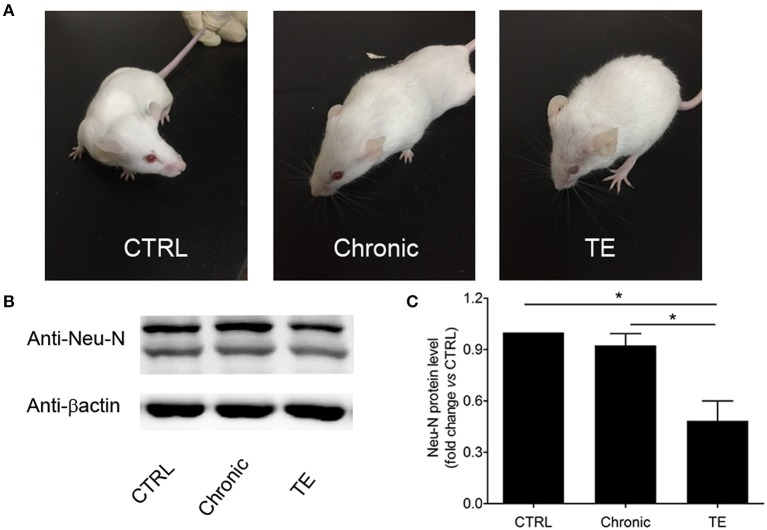
Establishment of a murine model of toxoplasmic encephalitis (TE) **(A)** and identification of neuron damage in the brain of mice with TE **(B,C)**. *P < 0.05.

The authors apologize for this error and state that this does not change the scientific conclusions of the article in any way. The original article has been updated.

